# Mammalian Hemopexin Protects Blood-Feeding Dipterans from Heme Toxicity and Promotes Parasite Transmission

**DOI:** 10.21203/rs.3.rs-10295862/v1

**Published:** 2026-10-23

**Authors:** Joel Vega-Rodríguez, Francis de Souza Saraiva, Thiago Alves e Silva, Octavio Talyuli da Cunha, Eva Iniguez, Patricia Alvarenga, Ana Barletta, Heather Kudyba, Mahnaz Minai, Motoshi Suzuki, Emanuela Tolosano, Derron Alves, Jesus Valenzuela, Carolina Barillas-Mury, Shaden Kamhawi

## Abstract

Blood feeding exposes arthropod vectors and the pathogens they transmit to toxic heme released during hemoglobin digestion. Although mammalian blood proteins can cross the mosquito midgut into the hemolymph, whether they remain biologically active within the vector is unknown. Proteomic analysis of *Anopheles gambiae* hemolymph identified mammalian proteins enriched by *Plasmodium* infection, including several involved in heme and iron metabolism. This enrichment was dependent on parasite invasion of the midgut epithelium. We show that mammalian hemopexin, a high-affinity heme scavenger, retains its heme-binding activity within mosquito tissues, where it mitigates heme toxicity, preserves midgut barrier integrity and microbiome homeostasis, and promotes *Plasmodium* development. These protective effects were lost with recombinant hemopexin unable to bind heme, establishing heme scavenging as the underlying mechanism. Hemopexin also promoted *Leishmania* parasite development in the sand fly and supported the fitness of *Aedes aegypti* and sand flies. Thus, parasite induced-redistribution of host hemopexin establishes a functional link between mammalian heme homeostasis, vector physiology, microbiome homeostasis and pathogen development, revealing a previously unrecognized host–vector–pathogen interaction and a potential vulnerability for transmission-blocking interventions.

